# Case Report: The first probable Hong Kong Chinese case of *LPIN1*-related acute recurrent rhabdomyolysis in a boy with two novel variants

**DOI:** 10.12688/f1000research.20343.1

**Published:** 2019-09-02

**Authors:** Sau Wing Yim, Tina Yee Ching Chan, Kiran M. Belaramani, Sze Shun Man, Felix Chi Kin Wong, Sammy Pak Lam Chen, Hencher Han Chih Lee, Chloe Miu Mak, Chor Kwan Ching

**Affiliations:** 1 Department of Paediatrics and Adolescent Medicine, Tuen Mun Hospital, Tuen Mun, Hong Kong; 2 Chemical Pathology Laboratory, Department of Pathology, Hong Kong Children's Hospital, Kowloon Bay, Hong Kong; 3 Kowloon West Cluster Laboratory Genetic Service, Chemical Pathology Laboratory, Department of Pathology, Princess Margaret Hospital, Hong Kong, Laichikok, Hong Kong; 4 Department of Chemical Pathology, Prince of Wales Hospital, Shatin, Hong Kong; 5 Chemical Pathology Laboratory, Department of Pathology, Queen Elizabeth Hospital, Kowloon, Hong Kong

**Keywords:** Hong Kong Chinese, LPIN1, Rhabdomyolysis, Novel variants

## Abstract

Recurrent rhabdomyolysis is frequently ascribed to fatty acid ß-oxidation defects, mitochondrial respiratory chain disorders and glycogen storage-related diseases. In recent years, autosomal recessive *LPIN1* mutations have been identified as a prevailing cause of severe rhabdomyolysis in children in Western countries. We report the first probable Hong Kong Chinese case of recurrent severe rhabdomyolysis in early childhood caused by *LPIN1* variants. Compound heterozygous novel variants NM_145693.2(LPIN1):c.[1949_1967dupGTGTCACCACGCAGTACCA]; [2410G>C] (p.[Gly657Cysfs*12];[Asp804His]) were detected. The former variant was classified as likely pathogenic while the latter variant was classified as a variant of uncertain significance (VUS) based on the guideline published by the American College of Medical Genetics and Genomics (ACMG) in 2015. Although the genetic findings were inconclusive, the patient’s presentation was compatible with LPIN1-related acute recurrent rhabdomyolysis, and the patient was treated as such. The early recognition, timely diagnosis and management of this condition are important to avoid fatal consequences. To our knowledge, there has been no previous report in the English-language literature of a child with Chinese ethnicity and *LPIN1*-related acute recurrent rhabdomyolysis (MIM #268200).  Functional characterization of the novel variants detected in this study are warranted in future studies.

## Introduction

Rhabdomyolysis is an uncommon but potentially fatal condition. It results from an acute muscle fiber breakdown resulting in the release of intracellular muscle constituents into the circulation. There are multiple causes for rhabdomyolysis. However, recurrent rhabdomyolysis is frequently attributed to metabolic myopathies. The differential diagnoses include fatty acid ß-oxidation defects (FAOD), mitochondrial respiratory chain disorders and glycogen storage diseases. When initial investigations including biochemical metabolic work-up are unremarkable, muscle biopsies for histological and enzymatic studies are commonly performed. In recent years, autosomal recessive mutations in *LPIN1* are being increasingly recognized as an important cause of acute recurrent rhabdomyolysis in childhood in the Western population, after the exclusion of FAOD, mitochondrial respiratory chain disorders and glycogen storage diseases. Lipin 1 (LPIN1), encoded by *LPIN1* gene, belongs to the LPIN gene family which also include *LPIN2* and *LPIN3*. Lipin 1 is predominantly expressed in skeletal muscle and adipose tissue ^1– 3^. Among lipins 1, 2 and 3, lipin 1 is the only lipin which is expressed significantly in muscles ^2, 4^. Lipin 1 is a Mg ^2+^-dependent phosphatidic acid phosphohydrolase (PAP) that catalyzes the dephosphorylation of phosphatidic acid to yield diacylglycerol and inorganic phosphate ^5^. It has also been found to exert transcriptional co-regulator activity ^6, 7^. The human *LPIN1* gene is mapped to chromosome 2p25.1 and contains 20 exons. Apart from acute recurrent rhabdomyolysis, *LPIN1* variants have also been found in adults with statin-induced myopathy, metabolic syndrome and type 2 diabetes mellitus ^8– 11^. We hereby report the first probable Hong Kong Chinese case of acute recurrent rhabdomyolysis in a boy with compound heterozygous *LPIN1* variants who presented with recurrent rhabdomyolysis since the age of 15 months.

## Case report

A 15-month-old boy was brought to the hospital complaining of coryzal symptoms for three days, and dyspnea and fever for one day. He was seen by a general practitioner a day prior to the admission and was given an intramuscular injection of an uncertain drug for his illness. After the injection, he complained of passing dark-colored urine twice. Physical examination showed good perfusion and the patient’s blood pressure was 113/65 mmHg. His body weight and height were at the 75 ^th^ percentile and 50 ^th^ percentile, respectively, for his age and sex. Respiratory examination revealed bilateral wheezes and the initial impression was acute bronchiolitis. Creatine kinase (CK) was markedly elevated to 127,494 U/L (reference interval: 39–308 U/L). Urine dipstick was positive for hemoglobin. After centrifugation of the urine through a 30 kDa microconcentrator membrane, urine dipstick for hemoglobin remained positive, confirming that the positive urine dipstick result without ultrafiltration was likely due to the presence of myoglobin, which cross-reacts with the peroxidase reaction employed by the urine dipstick for hemoglobin testing. Plasma creatinine and potassium were normal. Liver function tests revealed an elevated alanine aminotransferase (ALT) of 658 U/L (reference interval: 9–25 U/L) on the first day of admission; the value peaked on the second day of admission with a reading of 1,418 U/L. Apart from CK, lactate dehydrogenase (LDH) level was also raised to 9,154 U/L (reference interval: <350 U/L) and it peaked on the second day of admission at a value of 11,409 U/L. Cardiac troponin-I level was normal and electrocardiogram (ECG) did not show any abnormalities. A diagnosis of rhabdomyolysis was made.

Intravenous fluid was given in view of poor appetite and later tapered off as diet was better tolerated. CK came down to 10,909 U/L on the fourth day of admission. The boy had good urine output and there was no more passage of dark-colored urine since day three of the admission. Nasopharyngeal swab taken during admission was positive for respiratory syncytial virus. Dyspnea gradually resolved. On day five of illness, the CK, ALT and LDH had markedly improved. Repeated urine myoglobin and hemoglobin testing were negative. He was followed up two weeks later and was well then. There was no more passage of dark-colored urine.

Thereafter, he defaulted follow up and presented to us again at six years of age. This time, he complained of bilateral calf pain with antalgic gait associated with coryzal symptoms and fever. The episode was not preceded by any injuries nor excessive exercises. There was also no preceding administration of any intramuscular injection. Upon detailed history taking, the patient revealed that he frequently got muscle cramps after exercise. There was no family history of recurrent rhabdomyolysis, malignant hyperthermia or musculoskeletal diseases. The parents were both Chinese and non-consanguineous.

**Figure 1.**
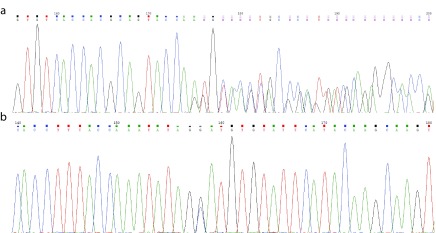
Electrophoretograms of the two novel *LPIN1* variants. ( **a**) NM_145693.2( *LPIN1*):c.[1949_1967dupGTGTCACCACGCAGTACCA] p.[Gly657Cysfs*12]. ( **b**) NM_145693.2( *LPIN1*):c.[2410G>C] p.[Asp804His].

Physical examination revealed bilateral calf tenderness in the absence of weakness. There was no associated hepatosplenomegaly. He was well built with a weight and height at the 50 ^th^ and 75 ^th^ percentile, respectively, for his age and sex. Investigations during this admission again revealed an elevated CK (27,442 U/L), LDH (658 U/L) and ALT (138 U/L). Urine for myoglobin and hemoglobin (analyzed by same methods as before) were both positive. Plasma creatinine and potassium were normal. Hydration was instituted and the CK levels normalized 10 days later. Due to the recurrent episodes of rhabdomyolysis, further investigations were undertaken including blood gas, plasma lactate and ammonia but they were all unremarkable. Metabolic screening was performed and showed a normal pattern of plasma amino acids, serum acylcarnitine profile and urine organic acid profile. Genetic analysis of all coding exons of *LPIN1* reviewed two heterozygous novel mutations: NM_145693.2(LPIN1):c.[1949_1967dupGTGTCACCACGCAGTACCA]; [2410G>C] (p.[Gly657Cysfs*12];[Asp804His]) ( Figure 1). The parents also underwent genetic tests for *LPIN1*. Each of the parents was a heterozygous carrier of one of the patient’s variants, which confirmed compound heterozygosity of the variants in the patient. The former variant was classified as likely pathogenic while the latter variant was classified as a variant of uncertain significance (VUS). Although the genetic findings were inconclusive, the patient’s clinical presentation was compatible with LPIN1-related acute recurrent rhabdomyolysis, and muscle biopsy was not done.

In spite of the uncertainty in the genetic findings, the likely diagnosis of LPIN-related acute recurrent rhabdomyolysis was conveyed to the family with proper genetic counseling conducted. Advice to avoid recurrent episodes of rhabdomyolysis, which include limiting strenuous exercise, ample hydration during exercise and prompt medical consultation in case of myalgia, was given. One year after the genetic diagnosis, he has been growing well and did not complain of passing any dark-colored urine, even during febrile illness.

The parents decided to have another baby and opted not for prenatal testing for *LPIN1* variants. Prenatal testing carries intrinsic risk to the fetus and, in our opinion, is not recommended for this disease which is amendable to treatment and not debilitating. Post-natal genetic test on the younger sister confirmed that she also carried the two familial heterozygous variants; upon this finding, counselling was given to the parents.

## Discussion

*LPIN1* mutations resulting in recurrent rhabdomyolysis was first described in three patients by Zeharia *et al*. in 2008 ^12^. In less than a decade since it was first described, there has been an increasing evidence in the literature that *LPIN1* mutations are one of the most frequent causes of recurrent rhabdomyolysis in childhood. Michot *et al*. reported that after excluding primary fatty acid oxidation disorders, recessive *LPIN1* gene mutations were found in 59% of the patients who exhibited severe recurrent rhabdomyolysis in childhood. The male to female ratio was reported to be 0.89. Out of the 17 cases described by Michot *et al*., more than half of them were Caucasian and the others were of African, Asian (Vietnamese) and Maghrebi ethnicity ^13^. Due to its relative novelty, the prevalence of the disease is still unknown. To our knowledge, there has been no previous report in the English-language literature of a child with Chinese ethnicity and *LPIN1*-related acute recurrent rhabdomyolysis (MIM #268200).

The pathophysiology of *LPIN1*-related recurrent rhabdomyolysis is not fully understood. Accumulation of lipid droplets is a common finding in muscle biopsy ^14^. The loss of PAP enzymatic activity, rather than a loss in transcription co-regulator activity, plays a major role in the pathogenesis of this disease as the loss of the PAP activity alone without a loss in transcription co-regulator activity was sufficient to cause rhabodomyolysis ^15^. Apart from the PAP activity responsible for triacylglycerol and phospholipid biosynthesis, it also serves as a transcriptional co-activator together with peroxisome proliferator activated receptor coactivator-1α ( *PPARA*) and peroxisome proliferator activated receptor gamma coactivator-1 alpha (PGC-1α) to regulate genes encoding fatty acid oxidation and respiratory chain enzymes ^6, 7^. Multiple genes are either up-regulated or down-regulated in myoblasts of lipin-1-deficient patients ^16^. Furthermore, various inflammatory inducers including lipopolysaccharides, zymosan and proinflammatory cytokines have been shown to repress *LPIN1* expression in adipose tissue and muscular tissue ^17, 18^. Inflammatory inducers are usually produced when the body is under catabolic stress and the resulting suppressed lipin 1 activity may lead to rhabdomyolysis. This hypothesis might explain the fact that the majority of the severe episodes are associated with catabolic states such as a preceding illness ^13^. Lipin 1 was also found to play in a role in autophagy of mitochondria as PAP activity is required for the maturation of autolysosomes and lipin 1 deficiency leads to accumulation of aberrant mitochondria ^19^. This may explain the finding of mitochondrial aggregates on ultrastructural examination of muscle tissue in some patients ^14^. Sarcoplasmic reticulum stress response was recently identified to be an important player in the pathogenesis of this disease by contributing to catastrophic *de novo* lipogenesis of both phospholipids and neutral lipids ^20^.

Disease-causing *LPIN1* mutations are scattered throughout the coding region ^14^. An intragenic deletion (c.2295-863_2410-27del) is frequently seen in Caucasian patients, possibly representing a founder effect ^13, 14^. In our patient, two heterozygous novel variants of *LPIN1* gene are detected: the 19-nucleotide duplication NM_145693.2( *LPIN1*):c.[1949_1967dupGTGTCACCACGCAGTACCA] (p.[Gly657Cysfs*12]) causes a frameshift with a premature termination codon and hence has deleterious effect; the novel missense variant, NM_145693.2( *LPIN1*):c.[2410G>C] (p.[Asp804His]), is found in the highly conserved LNS2 (Lipin/Ned1/Smp2) domain near the C terminus of lipin 1, which contains an important Mg ^2+^-dependent catalytic site responsible for the PAP activity ^21^. This amino acid residue is highly conserved. The variant is predicted to be deleterious ( SIFT), damaging ( PROVEAN) and probably damaging ( Polyphen-2) by *in silico* analyses. At the time of reporting, both variants are absent from controls in the Exome Sequencing Project, 1000 Genomes Project, Exome Aggregation Consortium and Genome Aggregation databases. NM_145693.2( *LPIN1*):c.[1949_1967dupGTGTCACCACGCAGTACCA] (p.[Gly657Cysfs*12]) was classified as a likely pathogenic variant (Criteria: PVS1 and PM2) and NM_145693.2( *LPIN1*):c.[2410G>C] (p.[Asp804His]) was classified as a variant of uncertain significance (VUS)(Criteria: PM2, PM3 and PP3) based on the American College of Medical Genetics and Genomics (ACMG) guideline published in 2015 ^22^. Functional characterization of the two detected variants is warranted to confirm their pathogenicity.

All patients with *LPIN1* mutations present with recurrent rhabdomyolysis triggered by febrile illness, prolonged fasting or anesthesia ^12, 13^. Michot *et al*. reviewed 29 cases with recurrent rhabdomyolysis, in which 17 patients were confirmed to harbor *LPIN1* mutations. For the patients who harbor *LPIN1* mutations, the first episodes of rhabdomyolysis occurred before the age of five years. The mean age of presentation was 21 months old and the earliest age of presentation was at five months of age. The number of recurrent episodes ranged from one to ten per patient ^13^. Our patient presented at 15 months of age and has so far experienced two episodes of rhabdomyolysis. As more than 50% of patients with recurrent rhabdomyolysis were found to have *LPIN1* mutations after exclusion of FAOD ^13^, genetic testing for *LPIN1* mutations should be performed prior to muscle biopsy if metabolic investigations are unremarkable. Muscle biopsy is an invasive procedure and require general anesthesia which may trigger rhabdomyolysis.

Michot *et al*. also noted that among the 29 cases of unexplained rhabdomyolysis they studied, all cases eventually confirmed to have *LPIN1* mutations had a CK level of >10,000 U/L ^13^. In our patient, the CK levels were 127,494 U/L and 27,442 U/L during the first and second episodes of rhabdomyolysis, respectively. This is in line with Michot’s findings. Therefore, one should be vigilant to look for *LPIN1* mutations in any young children presenting with rhabdomyolysis with a CK level of >10,000 U/L.

The management of rhabdomyolysis is mostly supportive by providing aggressive fluid replacement therapy to protect the renal function. Besides, close monitoring is also indispensable during fasting and anesthesia. As rhabdomyolysis episodes could be fatal, prevention of such events is of utmost importance. Parents should be well educated on the symptoms and precipitating factors of rhabdomyolysis. They should also be given an action plan when the child exhibits muscle pain or fever. In our case, proper education of the parents at diagnosis has helped in preventing further episodes of rhabdomyolysis. In a group of five patients with biallelic *LPIN* mutations, Pichler *et al*. proposed that high-caloric intake at periods of stress and intravenous glucose during episodes of rhabdomyolysis may decrease the number of rhabdomyolysis episodes and the duration of each episode by preventing catabolism ^22^. Further studies are required to confirm the efficacy and safety of this approach.

For *LPIN1*-related acute recurrent rhabdomyolysis, it was reported that mortality was as high as 30% during an acute episode of severe rhabdomyolysis ^14^. Between episodes, these patients thrived well. Intellectual disability was reported in one case at the age of nine years ^12^. Our patient has been normal between the episodes apart from frequent muscle cramps after exercise and there is no evidence of intellectual disability. However, further observation is needed in view of the young age of our patient. The long-term prognosis of this condition has been reported in a 25-year-old female with *LPIN1*-related recurrent rhabdomyolysis, who had bilateral common peroneal neuropathies in addition to a background residual distal myopathy detected one year following discharge from intensive care. It was uncertain whether the neuropathies were a result of critical illness, compression and/or severe weight loss during the admission or intrinsic to the underlying genetic lipin 1 deficiency ^23^.

Genetic confirmation of *LPIN1* mutations not only helps the patient but also their family members. In our case, the younger sister had genetic testing done soon after birth. This has raised the parents’ awareness of the disease and its preventive measures. The younger sister has been growing well and has had no episodes of rhabdomyolysis at the time of reporting at one year of age.

## Conclusion

This is the first probable reported case of *LPIN1*-related rhabdomyolysis in the Hong Kong Chinese population. It has been shown that *LPIN1-*related rhabdomyolysis is a major cause of severe rhabdomyolysis in early childhood so we should be vigilant of this condition. Screening for *LPIN1* mutations should be undertaken at an early stage in the work up of childhood rhabdomyolysis. As rhabdomyolysis in children has a high mortality rate, early recognition of the symptoms, timely diagnosis and proactive management are essential. Regarding prognosis, more observations and longitudinal studies are warranted to determine the long-term outcomes of this condition.

## Data availability

NCBI ClinVar: NM_145693.2( *LPIN1*):c.[1949_1967dupGTGTCACCACGCAGTACCA] (p.[Gly657Cysfs*12]). Accession number SCV000965694.

NCBI ClinVar: NM_145693.2( *LPIN1*):c.[2410G>C] (p.[Asp804His]). Accession number SCV000965695.

## Consent

Written informed consent for publication of the clinical details was obtained from the parents of the patient.
