# Supplementary material for: Case Report: The first probable Hong Kong Chinese case of LPIN1-related acute recurrent rhabdomyolysis in a boy with two novel variants
Source: F1000Res. 2019 Sep 2;8:1566. [Version 1] doi: 10.12688/f1000research.20343.1 (PMC6823901; doi:10.12688/f1000research.20343.1)
Supplement: Peer review history

# Peer review: Reviewer response for version 1

**DOI:** 10.5256/f1000research.22354.r54678

**Published:** 2019-10-31

**Authors:** Brian N. Finck

**Affiliations:** 1 Washington University in St. Louis, St Louis, MI, USA

Lipin 1 plays important roles in regulating intermediary metabolism by acting as a lipid phosphatase to synthesize diacylglycerol from phosphatidic acid. This is an important step in glycerolipid synthesis and a metabolic branch point. Mutations in lipin 1 in people lead to severe, recurrent rhabdomyolysis that can be debilitating or fatal. While these mutations are rare in the general population, they account for a significant proportion of acute paediatric rhabdomyolysis cases. In this case report, Yim and colleagues detail the identification of novel mutations in LPIN1 that are associated with rhabdomyolysis in a boy in Hong Kong. The report is well done and complete. The authors have done a good job of detailing the important literature on the topic. No changes are requested.

I confirm that I have read this submission and believe that I have an appropriate level of expertise to confirm that it is of an acceptable scientific standard.

# Peer review: Reviewer response for version 1

**DOI:** 10.5256/f1000research.22354.r53286

**Published:** 2019-09-16

**Authors:** Michael F. Buckley

**Affiliations:** 1 New South Wales Health Pathology, Prince of Wales Hospital, Randwick, NSW, Australia

This is the initial description of compound heterozygous variants in LPIN1 as a cause of rhadomyolysis in a person of Chinese ethnicity.

 The sequencing data provided is robust, the nomenclature used is correct and the ACMG classification for the missense change is conservative.  

 There is little in the way of improvements to the manuscript that I could suggest. The categorical statement "The human *LPIN1* gene is mapped to chromosome 2p25.1 and contains 20 exons" isn't correct as this gene has multiple transcription isoforms with different numbers and combinations of exons. The isoform used in this discussion certainly has 20 exons but other isoforms range up to 22 exons.

I confirm that I have read this submission and believe that I have an appropriate level of expertise to confirm that it is of an acceptable scientific standard.
